# Moral Challenges and Responsibilities in Caring for Relatives of Older Nursing Home Residents During the COVID-19 Pandemic in Sweden

**DOI:** 10.1007/s10728-025-00551-0

**Published:** 2025-11-27

**Authors:** Pier Jaarsma, My Eklund Saksberg, Therése Bielsten, Suzanne Cahill, Tiny Jaarsma, Petra Gelhaus

**Affiliations:** 1 Linköping University, Linköping, Sweden; 2 Jönköping University, Jönköping, Sweden; 3 Trinity College Dublin, Dublin, Ireland; 4 Region Östergötland, Linköping, Sweden

**Keywords:** COVID-19, Moral responsibilities, Nursing home nurses, Professional values, Relatives

## Abstract

Caring for relatives of older nursing home residents during COVID-19 was sometimes morally challenging for nursing home nurses. We identified four moral challenges: (1) providing versus withholding information, (2) respecting relatives’ wishes versus acting in accordance with professional standards, (3) acting in accordance with versus contrary to advance care plans, and (4) heeding versus ignoring visiting prohibitions. Care ethicist Margaret Urban Walker’s framework of moral responsibility together with values listed in the ICN code of ethics for nurses were used as points of departure for reflection on these moral challenges. Each challenge was described, and moral responsibilities were charted and discussed in terms of moral relationships between nursing home nurses and the relatives of older residents, nurses’ moral identity as a nurse, and nursing’s moral values as listed in the ICN code of ethics for nurses. Nursing home nurses’ moral responsibilities could be connected to many moral values of the nursing profession, such as empathy, responsiveness, caring, advocacy, equality, inclusivity, and compassion. However, these values have a limited effect on direct moral action, as different values can be addressed for opposite action alternatives.

## Introduction

Some moral challenges in a pre-COVID-19 context have been described, including communication and cooperation between nursing home nurses and relatives of residents [3, 4, 18]. It is well known that residents of nursing homes were particularly vulnerable during the COVID-19 pandemic with close physical contact with caregivers, and proximity to other residents as relevant factors [7]. In Sweden, there were high death rates among the elderly and an inquiry ordered by the state acknowledged that this was a consequence of an unprepared and ill-equipped care for the elderly [26]. The COVID-19 pandemic also changed the nature of nurses’ relationships with residents, and relatives of residents, which lead to ethical challenges for nurses [17]. Several studies reported problematic communication between nursing home nurses and relatives during the COVID-19 pandemic. Relatives reported that communication with nursing home staff who had limited Swedish language skills was challenging during visiting restrictions [8] taken by the government to protect nursing home residents against infections [15]. Nursing home nurses were sometimes instructed not to share information about an infected resident with other residents and their relatives [11]. Nursing home nurses also endured moral distress due to policies that prevented relatives from visiting the residents at the end of their life [19]. To address concerns relating to nursing home deaths and advance care planning during the COVID-19 pandemic and beyond, a relational and familial ethic has been advocated [20]. However, reports on the ethical challenges of nursing home nurses and care ethical reflections in relation to caring for relatives of older nursing home residents are limited. Yet knowledge about ethical challenges and the accompanying moral responsibilities is needed for nurses as well as for nursing students, so they can be prepared for similar circumstances in the future. This study aims to partly fill this gap by charting, analyzing and discussing moral responsibilities of nursing home nurses’ caring for relatives of older nursing home residents during the COVID-19 pandemic in Sweden (Table 1).

**Table 1:** Interview guide

1	Can you tell me about an event (concerning a resident) where in retrospect you think that the outcomes would have been better if the situation was handled differently?
2	Can you tell me about an event (concerning a resident) where in retrospect you think that the outcomes were the best possible and where you believe the situation was handled the best way possible?
3	Can you identify and describe an event (concerning a resident) during the COVID-19 pandemic where you prioritized differently compared to pre-COVID-19?
4	Can you identify and describe an event (concerning a resident) during the COVID-19 pandemic where you did not prioritize differently than before COVID-19?
5	Can you describe an event (concerning a resident) during the COVID-19 pandemic in which it was difficult to prioritize?
6	Can you describe an event (concerning a resident) during the COVID-19 pandemic in which it was easy to prioritize?
Follow-up questions (for each of the six questions):	
a	What led up to the event?
b	What were your experiences of the event itself?
c	What was the outcome of the event?
d	What factors contributed to your prioritization?

## Methods

### Data Collection

A qualitative interview study was performed using an inductive/deductive approach. Data was collected through in-depth interviews, namely, retrospective self-reports. Nursing home nurses were purposefully selected for these interviews. The respondents consisted of 21 registered nursing home nurses (Table 2). The interviews were conducted between February and May 2021. An interview guide with semi-structured open-ended questions using the critical incident technique [9, 23], to help respondents recall their experiences, was used (see Table 1). We identified and analyzed 58 events concerning residents, the results of which were published elsewhere [1]. One of those results, responding sensitively to relatives’ expectations, concerned relatives of residents. However, outside our dataset of 58 events, respondents noticeably also reported additional events concerning relatives of nursing home residents. These events, some of them accompanied by moral challenges, were outside the intentional object of the study, which focused on events concerning residents. We realized that some (or all) of the moral challenges primarily concerning relatives of nursing home residents were sufficiently interesting and important to share in the research community. We therefore chose to closely reread all of the transcripts of these interviews, to answer the following research questions: 1. What were nursing home nurses’ moral challenges in caring for residents’ relatives during the COVID-19 pandemic? and 2. What were nursing home nurses’ moral responsibilities pertaining to these challenges?

**Table 2:** Characteristics of the sample (n = 21)

Gender		
	Female	19
	Male	2
Age		
	21–30	5
	31–40	4
	41–50	6
	51–60	4
	> 60	2
Position		
	Registered nurse	21
Years of nursing experience		
	0–9	6
	10–19	8
	20–29	3
	30–39	2
	40 +	2
Responsibility		
	Older residents	6
	Residents with dementia	2
	Older residents and with dementia	13

### Analyses

In order to answer our first research question, a six-phase process of reflective thematic analysis was followed recursively and iteratively: (1) familiarization with the data; (2) generating initial codes; (3) generating themes; (4) reviewing potential themes; (5) defining and naming theme; (6) producing the report [6]. Table 3 shows the connection between codes and themes.

**Table 3:** Connection between codes and themes

Codes	Themes
Being the information center point at the beginning of the pandemic Informing relatives and explaining Prohibition to inform relatives about Covid-19’s presence Informing relatives about Covid-19’s absence in the nursing home	Providing versus withholding information
Relatives often do not understand focus should shift from cure to care and symptom alleviation Relatives are often pushy about which care they believe the resident should have Relatives demanding treatment Troublesome relatives criticizing everything	Respecting relatives’ wishes versus acting in accordance with professional standards
Not consulting relatives or residents about medical care plans Medical care plans were necessarily written without meeting relatives and the residents because of shortage of time Nurse's initiative to contact relatives about the appropriateness of hospital care	Acting in accordance with versus contrary to advance care plans
Taking care of relatives when a resident is dying Exception on the visiting prohibition when a resident was dying Relatives use PPE to visit a dying resident Relatives accepting PPE when visiting a resident at the end of life Informing and supporting relatives with close supervision on the PPE when visiting a dying relative Grateful relatives to be able to visit a dying resident which was not granted in the hospital The importance of a last farewell for relatives	Heeding versus ignoring visiting prohibitions

**Fig. 1:**
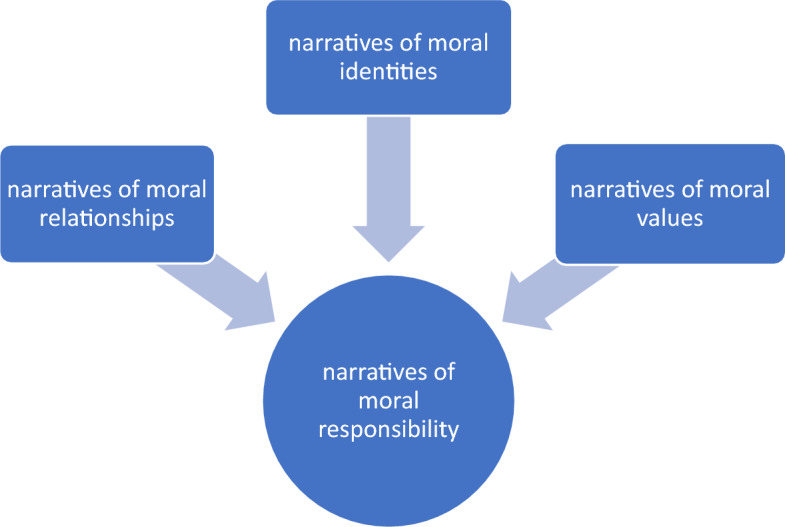
Narrative understanding of moral responsibility in terms of relationships, values, and identity

The theoretical point of departure for ethical reflection to deductively answer our second research question was the ethical framework of care ethicist Margaret Urban Walker [30, 31]. Walker theorizes moral responsibility in terms of stories or narratives. The roots of moral responsibility are moral relationships, moral identity, and moral values (Fig. 1). As our empirical material consists of narratives emanating from nursing fundamentally understood as a moral practice [24], in which caring relationships, and moral and professional identities and values are expressed, Walker’s approach is particularly relevant. Walker’s framework was instrumental in our preliminary charting and discussion of nurses’ moral responsibilities. Charting and discussing such responsibilities are meaningful, since caring is a moral practice, and responsibilities are characteristic of moral practices [31]. For each theme we focused on the moral challenge and reflected upon it in terms of moral responsibilities in terms of relationship, identity, and values:

A *narrative of relationship* is a story of the relationship's acquired content and developed expectations, its basis and type of trust, and its possibilities for continuation. […] The agent's own *narrative of moral identity* is a persistent history of valuation that can be seen in a good deal of what a person cares for, responds to, and takes care of. […] [The *narrative of moral values*] is a history of our shared understandings of what kinds of things, relationships, and commitments really are important, and what their relative importance is [31].

Each of these themes/moral challenges were not found in all interviews. Ideally there will be a number of instances of the theme across the data set, but for qualitative analysis there is no definitive answer to the question of what proportion of a data set needs to display evidence of the theme for it to be considered a theme [5]. For each theme we will give as accurately as possible a description of the moral challenge experienced by the nurse or nurses, followed by an ethical analysis of moral responsibility in terms of moral relationships, moral identities, and moral values. As source for potentially relevant values, we use the ICN code of ethics for nurses [13].

## Results

We identified four moral challenges of nursing home nurses caring for the relatives of older residents during the COVID-19 pandemic in relation to the following themes: (1) providing versus withholding information, (2) respecting relatives’ wishes versus acting in accordance with professional standards, (3) acting in accordance with versus contrary to advance care plans, and (4) heeding versus ignoring visiting prohibitions.

### Providing Versus Withholding Information

This theme summarizes the moral challenge for a nursing home nurse of having to keep relatives uninformed about the presence or absence of COVID-19 in the nursing home while desiring to inform or reassure relatives. Nurse P1 addressed a prohibition given by her superiors regarding passing on information to relatives about the presence or absence of COVID-19 in the nursing home.

Relatives called and asked how things were. Did we have any [COVID-19] infection in the accommodation? We were not allowed to tell [them] whether we had it or not. […] We had written down a few sentences on how to respond […] to certain questions, what we should say, from our superiors. […] It’s crazy. I thought so at the time too. It was like this: The relative cries and then you must answer like this and like that. (P1).

At a later stage during the COVID-19 pandemic, relatives were told that there was no COVID-19 in the nursing home:

What we weren't allowed to say then, maybe we can say now, but we don't know. I must take it on my own responsibility, but it happens that I tell relatives that we are proud that we have no infection here now and we are very keen not to get anything in. (P1).

Nurse P1 reported that she was not allowed to tell the relatives whether they had or did not have COVID-19 in the nursing home. The management’s demand may have harmed the relationship of trust between the nurses and the relatives. The nurse’s exclamation of *“It’s crazy. […] It was like this: The relative cries and then you must answer like this and like that”* indicates that she experienced the demand from the management as unnatural in a communication context. A natural response would have been to reassure terrified relatives and inform them truthfully. As such, the management’s demand was experienced as incompatible with the nurse’s caring identity and destructive to the relationship with the relatives. As a solution to this moral challenge of having to keep relatives in the dark about the presence or absence of COVID-19 in the nursing home while desiring to inform or reassure relatives, the nurse later decided to go against the management’s demand and inform relatives truthfully about COVID-19’s absence in the nursing home. In doing so, she chose caring for nursing home relatives’ needs and maintaining a good relationship with them, over loyalty to management, and at the same time she identifies with the nursing home on her own terms (being “proud”, and “keen not to get anything in”). However, nurse P1 was aware of her problematic choice and indicated that she was willing to bear responsibility. The nurse’s narrative clearly demonstrated values of empathy (for the relatives’ fears), responsiveness (to the relatives’ need to be informed or to be reassured), and caring (by the very act of reassuring relatives in case of COVID-19 absence in the nursing home), which were, among other values of the nursing profession, emphasized in the preamble of The ICN Code of Ethics for Nurses [13].

### Respecting Relatives’ Wishes Versus Acting in Accordance With Professional Standards

This theme summarizes the moral challenge for nursing home nurses when relatives request curative treatment for a resident, but the nurses themselves deem palliative care more appropriate. If such random requests are honored, then this may lead to inequality of care between patients. Nurses sometimes experienced differences of opinion with relatives about what is best for a resident. The disagreement centered around staff wanting palliative care for the patient and relatives wanting more aggressive treatment:

Many times, it is about situations where you may not really understand as a relative that your mother or father is very multi-diseased, perhaps suffering from dementia. […] It's basically that care turns to symptom relief and that you shouldn't keep investigating and undergo a lot of tedious examinations when the person in question probably won't be able to undergo, for example, an operation if you find something that would lead to it. So, it's more things like that, you can sometimes feel that you have a slightly different opinion about what is best for the patient. (P2).

Relatives were experienced as obtrusive in their demands for care:

Many are sick and elderly and it’s the ‘end of the line.’ You don't go to any price which is what some want...and when I talk about some [people], it's often relatives who are pushy, as it were. […] As long as they can get good information and understand why you make certain decisions or why you think the way you do, it usually goes well in the end anyway. And that it is in the best interest of the patient. (P2).

Nurse P2 reported that she sometimes experienced relatives to be badly informed about what is in the best interest of the patient, which resulted in their demanding curative treatments in cases where palliative care was more appropriate. She also asserted that when given the right information, relatives can be persuaded not to demand meaningless and burdensome treatments and examinations for the resident at the end of life. In her narrative, the vulnerability of the relationship between nurses and relatives becomes poignantly clear. The nurse talks about “pushy” relatives. The term ‘pushy’ has a negative connotation, and signals a (possible) deterioration in the relationship between the nurse and relatives, which is ethically challenging for the nurse. If the nurse and the relatives create a sufficiently trusting relationship to one another, an information deficit can be corrected and consent in the resident’s best interest can be achieved. Conflicts between nurses and relatives often revolve around hospitalization and the initiation of lifesaving treatment [21].

The narrative of responsibility of nurse P2 appears to center around the professional value of advocacy, i.e., “supporting others in speaking for themselves or speaking on behalf of others who cannot speak for themselves.” [13] In the nurse’s narrative, people who need nurses’ advocacy are people with multimorbidity or people with dementia at the end of life. They need to be protected from well-meaning relatives’ demands for meaningless but burdensome treatments and examinations at the end of life. Nurse P3 spoke about her experience of inequality of care triggered by relatives’ demands and their subsequent influence on the quality of care. In this instance, the nurse felt palliative care to be more appropriate, but the relatives wanted staff to continue with curative medical treatment of the resident. The latter request was honored, leading to the ethical problem of inequality between patients:

I have a patient. She turns 100 this year. And she is one of those people who has had a lot of infections. […] She had difficulty answering what she wanted herself because she was so confused right then. But the relatives really wanted you to do everything…or everything...but that we would try to treat her so that she received antibiotics and more medications. But I think that it would probably be individual. […] If it had been a different patient, then surely, they wouldn't have done so much. (P3).

Inequality was created when relatives’ wishes *“to do everything”* for a resident were respected, but *“if it had been a different patient”* [with less persisting relatives or no relatives at all], *“then surely, they wouldn't have done so much (P3).”* Inequality in nursing may be due to differences in residents’ social backgrounds, such as the social standing of the patients themselves or their relatives [29]. In our study, inequality was related to whether residents had or did not have persisting relatives present to represent their interests.

Particularly relevant for this moral challenge is Article I of the Universal Declaration of Human Rights (UDHR): “All human beings are […] equal in dignity and rights [28].” Nurse P3’s narrative of *“if it had been a different patient, then surely, they wouldn't have done so much”* suggests inequality in both dignity and rights. So, the nurse’s responsibility, in line with the UDHR and as suggested by the ICN code of ethics for nurses, lies in promoting an environment in which this human right of equality in dignity and rights is acknowledged and respected.

### Acting in Accordance with Versus Contrary to Advance Care Plans

This theme summarizes the moral challenge for nursing home nurses to act in accordance with an advance care plan knowing that it had been drawn up without inclusion of residents and relatives. Nurse P16 spoke of contacting relatives to inquire about their wishes regarding hospitalization. The advance care plan had been drawn up advising against hospitalization, but without seeking consent from relatives or residents. The nurse criticizes this and reports having no regrets about acting contrary to such an advance care plan:

A doctor had written that this patient [with dementia] should not be sent to hospital but should get palliative care. But I thought that those care plans didn't feel quite right because I knew the background. They were drawn up without consent from relatives or patients. So, I contacted the relative. […] This patient unfortunately died in the hospital a week later, but I find it hard to imagine that I would have done anything other than contact the relative and put the decision in [his or her] hands as to whether to hospitalize or not. (P16).

Advance care planning—with inclusion of residents and relatives—is an important element in decision-making in nursing homes [2, 22]. In our study, respondent P16 narrates that advance care plans during the COVID-19 pandemic had been drawn up without such discussions. This nurse appears to find this lack ethically challenging: *“I thought that those care plans didn't feel quite right because I knew the background. They were drawn up without consent from relatives or the patient.”* Therefore, the lack of inclusivity appears to detract from respondent P16’s identity as a professional nurse responsive to the needs of relatives, including the need to be included in advance care plan discussions. Moreover, such inclusion will promote good relationships with relatives and conversely, lacking such inclusion risks detracting from good relationships between nurses and relatives. Inclusivity and responsiveness are recognized as professional values, among others, in the ICN code of ethics for nurses [13].

Respondent P16 took the responsibility upon themselves to challenge the advance care plan, by contacting the resident’s relatives and *“put[ting] the decision in [his or her] hands as to whether to hospitalize or not”,* thereby seemingly overruling the physician who wrote the advance care plan. Interestingly, the nurse seems to understand that the relatives have the right to make the decision regarding hospitalization. This is not the case in Swedish healthcare. Usually, it is a responsible doctor at the hospital who makes that decision, and advance care planning is meant to allow better decision-making with sufficient background knowledge about the patient’s overall health situation regarding the patient’s values and with the inclusion of relatives. At first sight, the nurse’s action appears to be at odds with the professional values of collaboration, judgement, competence, and safety [13]. Nevertheless, the poor quality of advance care plans under pandemic conditions resulted in a lack of trust among professionals and made their intended usefulness in this case obsolete.

### Heeding Versus Ignoring Visiting Prohibitions

This theme summarizes the moral challenge for nursing home nurses to let relatives strictly adhere to rules of visiting prohibition during the COVID-19 pandemic to protect themselves, the residents, their relatives and the wider public or to allow relatives to visit a dying resident, while trying to minimize the risk of infection. Nurses P11 and P14 spoke of allowing relatives to visit a dying resident despite visiting prohibitions:

We have had patients who have been dying and then when there was a visiting prohibition, we still tried [to allow] one or two people to come in. (P11).

We've done it anyway, even if there was a visiting prohibition and someone got sick, their relatives were allowed in […] As I said to a relative or on an occasion ‘when someone is very ill and at the end of life there is no turning back’. (P14).

Nurse P15 narrated that when relatives visited a dying resident, the nurse supported, informed, and explained to relatives about basic hygienic routines and Personal Protective Equipment (PPE). There was close supervision during the visits and relatives accepted PPE after explanation of PPE’s importance:

The relatives were very grateful that they were informed that they could come, with protective equipment. I explained basic hygiene routines a bit so that they still felt safe and could sit in there for the last time. (P15).

It was stressful to take protective equipment on and off every time you entered this room, and you knew that there were frequent inspection visits and that you had to support relatives and help them to understand why they had to wear all this protective equipment when they sat with the patient. (P11).

Relatives showed their gratefulness for being allowed to visit a resident at the end of life despite a visiting prohibition:

The relatives were very grateful that they were allowed to come, because in the hospital they had not been allowed to visit him while he was there. There was a strict visiting prohibition there. (P11).

At the beginning of the COVID-19 pandemic, the World Health Organization recommended that access to visitors in nursing homes should be restricted and avoided as much as possible [10, 32]. Some nurses in our study implicitly narrated that they did not follow the recommendation from the WHO when they judged a resident to be at the very end of life. The relatives were allowed in, so that they “*could sit in there for the last time (P15).”* As one respondent explained: *“when someone is very ill and at the end of life there is no turning back (P14),”* meaning that there will be no other opportunity for the relatives to visit the resident. In the nurses’ first action alternative, they appear to care about and for the relationships between relatives and residents until the very end of the residents’ life, demonstrating values of the profession such as respect, empathy, responsiveness, and compassion [13]: compassion for the relatives, emanating from empathy for the relatives’ plight under the tragic circumstances of the COVID-19 pandemic; responsiveness to the relatives’ needs; and respect for the resident’s dignity at the end of their life. This action alternative is compatible with a person-centered approach and appears to be in line with their identities as professional nurses. The relatives were “grateful”, which means they realized the moral conflict and were glad that their needs weighed on the nurses more heavily than did isolation during the pandemic. However, this action alternative risked visitors, other residents, society at large, and the nurses themselves with being infected with COVID-19, which at that time was a severely incapacitating and possibly lethal virus.

The nurses’ second action alternative, i.e., refraining from letting relatives visit a dying resident—can be supported by the values of public good, responsibility, collaboration, solidarity, trust, and safety, also listed as professional values of the nursing profession [13]. The nurses are responsible not only for the relatives and for themselves, but also for the public good, i.e., they must express solidarity with the wider public and care about and for their safety. Trust as a professional value plays a role because “the wider public also needs to be able to trust a nurse to take their interests into account by preventing COVID-19 from further spread” [14]. Collaboration is a relevant value for the second action alternative, when nurses collaborate with those who issued the recommendation.

The narratives from respondents P11 and P14 appear to show that they weighed both action alternatives in this moral challenge. P11 states that *“there was a visiting prohibition, [but] we still tried to let one or two people come in”,* and P14 states that *“we've done it anyway, even if there was a visiting prohibition and someone got sick, their relatives were allowed in.”* In choosing the first action alternative, the nurses seem to act in accordance with a person-centered ethics.

## Discussion

Structural shortcomings in the care of older people in Sweden, such as the need for higher staffing levels, greater expertise, and reasonable working conditions [26], mitigate nurses’ moral responsibilities. With this caveat, and legal accountabilities aside, we reflected upon the moral responsibilities of nurses for the above-described ethical challenges, considering the International Council of Nurses’ code of ethics for nurses and using Margaret Urban Walker’s framework for charting these moral responsibilities [30, 31].

Nurses’ moral responsibilities were multifaceted and could be connected to many moral and nursing professional values. Narratives of relationships—mainly between nurses and relatives, and narratives of identities—mainly nurses’ professional identities—were used to explain nurses’ moral responsibilities. Margaret Urban Walker’s approach could successfully highlight ethical motivations and deliberations that support practical decision-making.

Our findings also reflect nurses’ moral attitudes and ethical considerations under the challenge of changed rules and organization in the COVID 19 pandemic. Rules that directly oppose classical care responsibilities, such as truthfulness and empathic communication risk being neglected, however important, justified, and useful they may be. Even the values of the ICN code of ethics for nurses have a limited effect on direct moral action, as different values can be addressed for opposite action alternatives. To make ethically sound decisions, every moral agent needs practical wisdom, which implies solid, comprehensive, and on-going ethical education. At present, ethics education for nurses in Sweden is primarily based on person-centered ethics [16]. In times of crisis, such as during the height of the COVID-19 pandemic, consequentialism-inspired arguments may need to be considered to reach ethically sound decisions. Broad ethical reflection, including care ethics, principles, duties, virtues, and consequences, is required on all levels of healthcare, and should be included in all healthcare education. Nurses must be equipped to confidently manage moral challenges, especially those that come with caring for patients and relatives of patients. Therefore, high-quality ethics pedagogy should be an essential component of nursing education [12]. Moreover, workplace ethics education may contribute to moral resilience, which may mitigate stress associated with moral challenges [25]. Regarding the ICN code, which incorporates all the key nursing values but can also be used eclectically, it would be useful to have guidelines on how values can be used and weighed in circumstances of scarcity and crisis.

Although our qualitative data technique does have weaknesses, our findings do appear to be transferable to relevantly similar situations (e.g., a new pandemic endangering vulnerable populations such as older nursing home residents). Limitations of our study are potential biases related to how we framed the interview questions using the critical incident technique [9, 23] and our choice of vocabulary; the respondents’ ability to recall events accurately; the respondents’ willingness to give what the interviewer wanted to hear (through the use of leading questions); and which respondents were willing to be interviewed in the first place [27].

## Conclusion

Nursing home nurses sometimes face moral challenges in communication, or the lack thereof, with relatives of nursing home residents. Moral responsibilities in the context of these moral challenges were discussed considering moral and professional relationships and identities and could be connected to many moral values of the nursing profession, such as empathy, responsiveness, caring, advocacy, equality, inclusivity, and compassion. However, these values have a limited effect on direct moral action, as different values can be addressed for opposite action alternatives.

## Data Availability

No datasets were generated or analysed during the current study.
